# miRNA164-directed cleavage of *ZmNAC1* confers lateral root development in maize (*Zea mays* L.)

**DOI:** 10.1186/1471-2229-12-220

**Published:** 2012-11-21

**Authors:** Jing Li, Guanghui Guo, Weiwei Guo, Ganggang Guo, Dan Tong, Zhongfu Ni, Qixin Sun, Yingyin Yao

**Affiliations:** 1 State Key Laboratory for Agrobiotechnology and Key Laboratory of Crop Heterosis and Utilization (MOE) and Key Laboratory of Crop Genomics and Genetic Improvement (MOA), Beijing Key Laboratory of Crop Genetic Improvement, China Agricultural University, Beijing, 100193, China; 2 National Plant Gene Research Centre (Beijing), Beijing, 100193, China; 3 Department of Plant Genetics & Breeding, China Agricultural University, Yuanmingyuan Xi Road No. 2, Haidian District, Beijing, 100193, China

**Keywords:** Maize *ZmNAC1*, miR164, Lateral root

## Abstract

**Background:**

MicroRNAs are a class of small, non-coding RNAs that regulate gene expression by binding target mRNA, which leads to cleavage or translational inhibition. The NAC proteins, which include NAM, ATAF, and CUC, are a plant-specific transcription factor family with diverse roles in development and stress regulation. It has been reported that miR164 negatively regulates *NAC1* expression, which in turn affects lateral root development in *Arabidopsis*; however, little is known about the involvement of the maize NAC family and miR164 in lateral root development.

**Results:**

We collected 175 maize transcripts with NAC domains. Of these, 7 *ZmNACs* were putative targets for regulation by miR164. We isolated one gene, called *TC258020* (designated *ZmNAC1*) from 2 maize inbred lines, 87-1 and Zong3. *ZmNAC1* had a high expression level in roots and showed higher abundance (1.8 fold) in Zong3 relative to 87-1, which had less lateral roots than Zong3. There was a significant correlation between the expression level of *ZmNAC1* and the lateral root density in the recombinant inbred line (RIL) population. Transgenic *Arabidopsis* that overexpressed *ZmNAC1* had increased lateral roots in comparison to the wild type. These findings suggest that *ZmNAC1* played a significant role in lateral root development. An allelic expression assay showed that trans-regulatory elements were the dominant mediators of *ZmNAC1* differential expression in 87-1 and Zong3, and further analysis revealed that miR164 was a trans-element that guided the cleavage of endogenous *ZmNAC1* mRNA. Both mature miR164 and miR164 precursors had higher expression in 87-1 than Zong3, which was the opposite of the expression pattern of *ZmNAC1*. Additionally, the allelic assay showed that the cis-regulatory element most likely affected *Zm-miR164b*'s expression pattern. A β-glucuronidase (GUS) assay showed that the *Zm-miR164b* promoter had higher GUS activity in 87-1 than in Zong3. In addition, we detected miR164b expression in the RIL population, and the results indicated that miR164b had a higher expression level in the RILs containing 87-1 promoter than those containing Zong3 promoter.

**Conclusion:**

Our results indicate one possible pathway in maize by which differences in *miR164b* promoter activity resulted in a different expression pattern for mature miR164 which negatively regulates *ZmNAC1* expression in 87-1 and Zong3, thereby contributing to a significantly different lateral root phenotype.

## Background

*NAC* genes that encode plant-specific transcription factors influence a diverse set of developmental processes. The NAC family proteins contain a consensus sequence known as the NAC domain (petunia NAM and *Arabidopsis* ATAF1, ATAF2, and CUC2) [1,2], which is located in the N-terminal region, but their C-terminal sequences are divergent in both length and amino acid sequence [3]. NAC proteins make up a large family, with a total of 109 and 140 predicted proteins identified in *Arabidopsis* and rice, respectively [4,5]. Members of the NAC family seem to play different roles in plant development. For example, *NAM* from petunia [1] and *CUC2* of *Arabidopsis*[2] (the first reported *NAC* genes) are involved in shoot apical meristem (SAM) development. By contrast, the *Arabidopsis NAC* gene *CUC3* has been reported to contribute to the establishment of the cotyledon boundary and the shoot meristem [6]. Another *Arabidopsis NAC* gene called *NAP* (NAC-LIKE, activated by AP3/PI) has been characterized as the target of two MADS box genes, *APETAL3* and *PISTILLATA*, which control cell division and cell expansion in stamens and petals [7]. *AtNAC1* has been characterized as an intermediary in the auxin-signaling pathway that activates genes encoding molecules involved in the specification of lateral root initiation [8]. In addition, NAC proteins have been implicated in defense and abiotic stress responses. Some ATAF subfamily genes, including *StNAC* from tomato and *AtAF1-2* from *Arabidopsis,* have been shown to be induced by pathogen attack and wounding [9]. More recently, some *NAC* genes such as *BnNAC* from *Brassica*[10], *AtNAC072* (RD26), *AtNAC019* and *AtNAC055* from *Arabidopsis*[11], and *SNAC1*[12] and *SNAC2*[13] from rice were found to be involved in the plant's response to various environmental stresses, including drought, salinity, and/or low temperature.

MicroRNAs (miRNAs) are a class of small, non-coding RNAs that regulate gene expression by guiding target mRNA cleavage or translational inhibition [14-16]. In *Arabidopsis*, miR164 can target five NAC domain-encoding mRNAs, including the *NAC1, CUC1, CUC2, At5g07680,* and *At5g61430* mRNAs [17]. *NAC1* is able to transmit auxin signals that promote lateral root emergence, and miR164 guides the cleavage of *NAC1* mRNA, which is followed by a mechanism to cleave *NAC1* mRNA and downregulate auxin signals [8,18]. Other groups have independently reported evidence for the miR164-mediated regulation of *CUC1*[17] and *CUC2*[19,20], which have been implicated in meristem development and the separation of aerial organs.

The process of lateral root (LR) development has been extensively studied in flowering plants and gymnosperms, and LRs begin in a specialized cell layer (called the pericycle) in the primary root (PR) [21,22]. In *Arabidopsis* and most other dicots, LRs are only formed from the pericycle cells that overlay the developing xylem tissue (the xylem pole pericycle). In other species, particularly cereals such as maize, rice and wheat, LRs arise specifically from the phloem pole pericycle, with additional contributions from the endodermis [23,24]. In *Arabidopsis,* a large body of evidence has indicated that auxin plays a pivotal role in lateral root development. Lateral root initiation begins with auxin-induced IAA14 degradation. This step allows the activation of *ARF7* and *ARF19* transcription factors, which then activate the expression of *LBD/ASL* genes. The LBD/ASL proteins in turn activate cell cycle genes and cell patterning genes, allowing the formation of a new lateral root primordium (LRP) [25,26]. In addition, auxin activates the transcription of *NAC1*, which upregulates the expression of two downstream auxin-responsive genes including *DBP* and *AIR3* to stimulate LR initiation [8]. The post-transcriptional and post-translational regulation of NAC1 was also reported as follows: the role of miR164 expression in late auxin response was intended to clear *NAC1* mRNA, which would attenuate the auxin signaling that inhibits lateral root development [18]. A RING-finger ubiquitin E3 ligase called SINAT5 promoted NAC1 ubiquitination and its subsequent degradation in order to attenuate the auxin response [27].

In this study, a miR164-targeted NAC domain gene that was designated *ZmNAC1* was isolated from maize. It was demonstrated that *ZmNAC1* overexpression in *Arabidopsis* leads to increased number of lateral roots. Further study showed that miR164 is one of the trans-acting factors that negatively regulates *ZmNAC1*, resulting in a different *ZmNAC1* expression pattern between the two inbred lines 87-1 and Zong3, contributing to a significant difference in the lateral root phenotype between these two lines. Moreover, our analysis also indicated that the promoter variation of maize *pri-miR164b* in 87-1 and Zong3 might be one in which the dominant cis-element affects the expression of *pri-miR164b,* leading to differences in mature miR164 expression.

## Results

### Identification of putative miR164-regulated NAC genes in maize

Putative NAC proteins in maize were identified using the conserved NAC domain sequences in a query that was performed by TBLASTN search. Predicted amino acid sequences of the full-length maize cDNA (PlantGDB) were used for the analysis. In this study, a total of 175 putative maize NAC proteins were obtained, and 105 NACs from *Arabidopsis* were used to construct the polygenetic tree. These NAC proteins could be classified into 3 groups and 14 subgroups on the basis of their predicted NAC domain amino acid sequences (Additional file 1). It has been reported that miR164 directs the regulation of 5 target NAC-domain transcription factor mRNAs in *Arabidopsis*[17]. To obtain putative miRNA-regulated *NAC* genes from maize, we searched the reverse complementary site for the mature miR164 in the 175 *ZmNAC*s. Maize miR164 sequences were identified in http://www.mirbase.org/. Seven *ZmNAC* genes, *TC258020*, *Zm390255026*, *Zm029753*, *Zm020717*, *Zm020987*, *Zm4253255028* and *Zm017452*, were found to be putative miR164 target genes (Additional file 2), and are shown in pink dots in supplemental Figure 1 (Additional file 1). Among these genes, the amino acid sequence corresponding to three of them, *Zm020987*, *TC258020* and *Zm017452,* showed high homology to *Arabidopsis* NAC1 protein (Additional file 1).

### Expression of putative miR164-regulated *ZmNAC* genes

**Figure 1:**
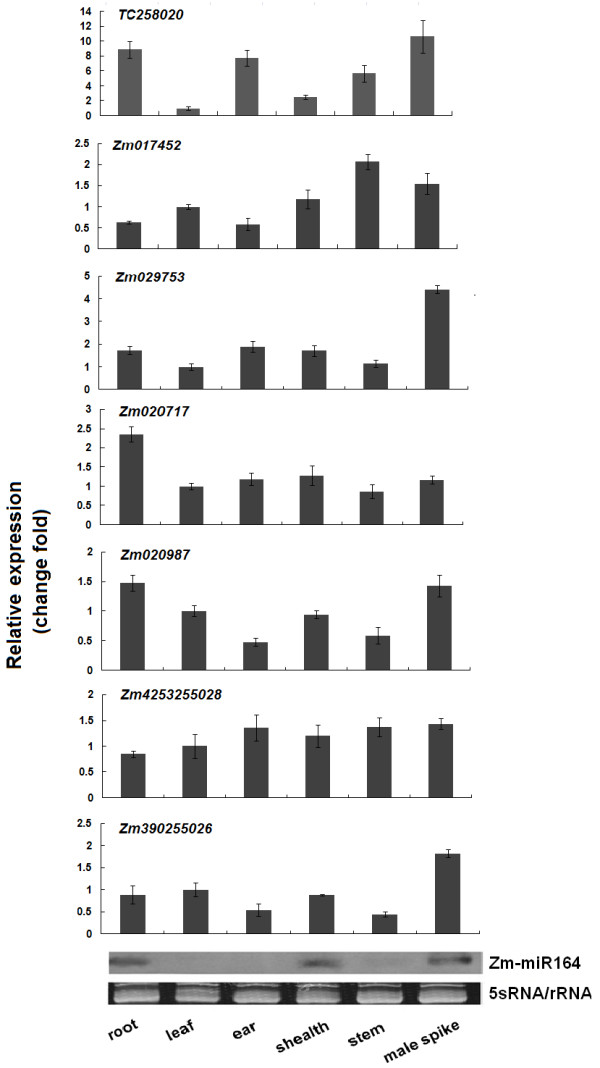
**Expression analysis of ZmNAC genes and miR164 in various maize tissues, including roots, leaves, ears, leaf sheaths, stems, and male spikes, as determined by real-time PCR.** The expression levels of each NAC gene in leaf was chosen as the calibrator. To perform the miR164 expression analysis, an RNA gel blot containing low-molecular-weight RNA (10 μg low molecular weight RNA per lane) was hybridized with an end-labeled DNA oligonucleotide that was complementary to miR164.

To assay the expression patterns of seven maize NAC genes (tc258020, Zm017452, Zm029753, Zm020717, Zm020987, Zm4253255028 and Zm390255026) containing the miR164 complementary site, six maize tissues from the roots, leaves, leaf sheaths, male spikes, ears and stems were used for real-time PCR. Because these genes showed a high degree of sequence conservation in the NAC domain, specific PCR primers were designed for the C-terminal region. The results indicated that *TC258020* and *Zm020717* had higher expression levels in their roots than in other analyzed tissues, whereas *Zm020987* and *Zm390255026* showed a lower expression level in the ears and stems than in other analyzed tissues (Figure 1). *Zm029753* and *Zm4253255028* accumulated in all organs and developmental stages (Figure 1). It has recently been proposed that miR164 guides the cleavage of NAC mRNAs in *Arabidopsis*[18]. An RNA gel blot of maize miRNA164 showed that the miR164 expression levels were higher in roots, leaf sheaths and male spikes than in other organs (Figure 1). This pattern was similar to the pattern of miR164 expression in *Arabidopsis*, where miR164 accumulated more in its roots and inflorescences than in other tissues [18]. The higher miR164 expression in roots suggested that miR164 might target the NAC gene in roots in vivo.

### The *ZmNAC1* gene and its expression in maize roots

**Figure 2:**
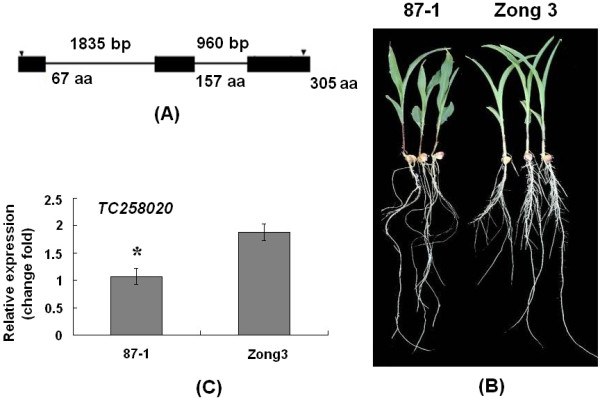
**The *ZmNAC1 *gene and its expression patterns.A**. Structure of the *ZmNAC1* gene. The first intron is 1835 nucleotides in length, with the 5^′^splice junction at cDNA position 523 bp; the second intron is 960 nucleotides in length, with the 5’splice junction at position 794 bp. The boxes and lines depict the exon and intron sequences, respectively. The arrowhead represents the translation start and stop site. **B**. The root phenotype of 6-day-old seedlings from 87-1 and Zong3. **C**. Expression analysis of *ZmNAC1* for inbred line 87-1 and Zong3 by real-time PCR.

In *Arabidopsis*, miR164 directs *NAC1* mRNA cleavage, which affects lateral root development [18]. We first sought to identify the putative miR164-targeted *NAC* genes in maize, which might be involved in lateral root development. Among the seven candidate miR164 target genes, *TC258020* encoded a protein of 305 amino acids and shared a high homology with *Arabidopsis NAC1*, so we named this gene *ZmNAC1*. The full-length 1657 bp cDNA of *ZmNAC1* was obtained by using rapid amplification of cDNA ends (RACE). The N-terminal residues contained the five conserved homologous blocks that characterize the NAC family. The divergent C-terminus displayed no homology to other known proteins. Our analysis detected a putative bipartite nuclear localization signal sequence (NLS) between amino acids 121 and 138 (Additional file 3). Based on the published maize genome sequences, *ZmNAC1* was mapped on chromosome 5 and has two introns within its coding region (Figure 2A).

*Arabidopsis NAC1* has been shown to play an important role in lateral root development. To determine the putative biological function of *ZmNAC1* in maize root development, the inbred lines 87-1 and Zong3 along with a set of RIL (recombination inbred lines derived from the cross between Zong3/87-1) were used, and in this population, either two inbreeds or the RILs showed a significant difference in their lateral root density. The lateral root density of 87-1 and Zong3 were 4.13 and 6.82 respectively (Figure 2B p < 0.01) and a large amplitude of variation in lateral root density (from 2.3 to 12.6) was observed among the RILs (Additional file 4).

The accumulation of *ZmNAC1* transcripts in roots among the inbred lines Zong3, 87-1 and their RILs was determined by real-time PCR, which revealed that *ZmNAC1* showed 1.8-fold (P < 0.05) higher expression in Zong3 than in 87-1 (Figure 2C) and had different expression levels among different RIL lines with a maximum of 1.41-fold higher than Zong3 and 3.04-fold lower than 87-1 (Additional file 5). To determine whether the lateral root phenotypes in RIL populations were associated with *ZmNAC1* expression level, the correlation coefficient between the lateral root density and the expression levels of *ZmNAC1* was calculated. These results showed that the correlation coefficient value reached 0.41 (P < 0.01), suggesting that differential *ZmNAC1* expression contributed 16.8% to the lateral root phenotype. Based on these results, we argued that *ZmNAC1* played important roles in maize lateral root development and contributes to the lateral root number difference between the inbred lines Zong3 and 87-1.

### Overexpression of *ZmNAC1* in *Arabidopsis* positively regulated lateral root development

**Figure 3:**
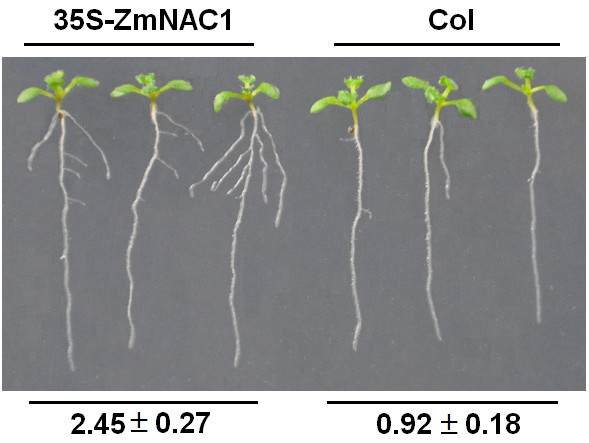
**Effects of *ZmNAC1 *overexpression on lateral root development.** Wild-type Columbia ecotype (Col) and transgenic seedlings overexpressing *35S-ZmNAC1* were grown on MS medium with 3% sucrose. Plants were photographed 11 d after germination. The numbers of lateral roots per centimeter of primary root of each seedling (average±SE) for wild type Col and *35S-ZmNAC1* are presented at the bottom (n=18). It can be clearly observed on the vertically oriented plates that the transgenic plants had many more lateral roots than the wild type.

To further confirm the biological function of *ZmNAC1*, we generated transgenic *Arabidopsis* plants that overexpressed the *ZmNAC1* gene. The transgenic plants with *35S: ZmNAC1* had a phenotype that displayed earlier and more lateral roots than the wild type. At 9 days after germination, the seedlings of *35S: ZmNAC1* transgenic lines (but not the wild type seedlings) developed lateral roots. At 11 days after germination, we measured the number of lateral roots per centimeter of primary root in *35S: ZmNAC1* and wild type plants, and the results showed that the lateral root density in the overexpression line and the wild type reached 2.45±0.27 and 0.92±0.18, respectively, with a significant difference between them (n=18, P < 0.01, *t*-test) (Figure 3). However, there was no difference in phenotype in the growth of the above-ground portion between the wild type and transgenic plants.

### *ZmNAC1* allelic expression between maize hybrids and parents

**Figure 4:**
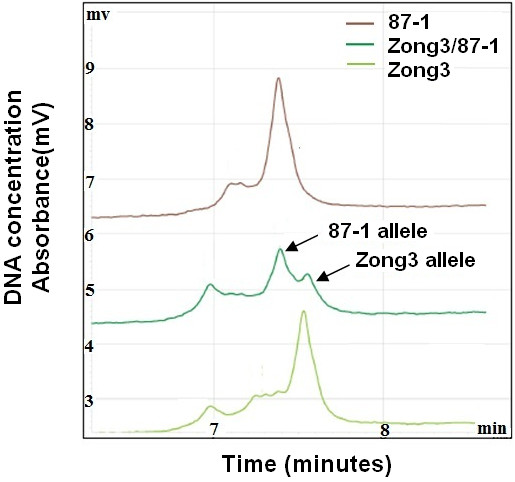
**RT-PCR and allele-specific cDNA quantification analysis with the WAVE dHPLC system.** The parental alleles of the *ZmNAC1* gene were cloned and sequenced, and an allelic polymorphism that had 4 bp less in the 5^′^UTR region of 87-1 than Zong3 was found. RT-PCR was then performed using primers designed for the conserved region between the alleles that encompassed the 4 bp Indel region. The RT-PCR products were separated and quantified using a WAVE dHPLC system. The longer DNA fragments that corresponded to the Zong3 allele had a higher affinity and therefore took a longer time to be eluted from the WAVE column than the shorter DNA fragments of the 87-1 allele eluted earlier. The x-axis shows the time in minutes when the DNA fragments were eluted. The y-axis demonstrates the UV absorbance that was used to measure DNA concentration or expression level. This analysis quantifies the allele-specific transcript as a relative ratio and does not measure the absolute transcription level.

Gene regulation involves numerous molecular interactions. In general, both cis-regulatory elements and trans-regulatory factors can play important roles. To determine whether the expression of *ZmNAC1* is regulated by a cis-, trans-acting or both mechanism, we compared the allele-specific expression of *ZmNAC1* in Zong3, 87-1 and their hybrid (Zong3/87-1), which was used as an excellent system to assay allele-specific expression. The two alleles from Zong3's female parent and 87-1 's male parent were compared in the hybrid Zong3/87-1 and were found to be equally affected by genetic background or environmental factors. The allele from Zong3 had a 4 bp insertion in the 5′UTR region compared to the allele from 87-1, which made it possible to distinguish the alleles from the two inbred lines and their hybrid; therefore, the parental transcript accumulation in the two inbred lines and the hybrid was examined using allele-specific RT-PCR analysis (Figure 4).

Different types of regulatory divergence can be detected experimentally by assaying the allele-specific gene expression in the two parents and the hybrid. When the alleles differ in their expression to the same extent in the parental plants as in the hybrids, then cis-acting genetic differences may be inferred [28,29]. When the alleles differ in their expression to a larger extent in the parental species than in the hybrids, then trans-acting genetic differences may be inferred. The WAVE dHPLC system was used to calculate the P-ratio, which is the ratio of allelic expression in the two parental lines, as well as the H-ratio, which is the ratio of alleles from the two parents in the hybrid. Then, we examined whether two *ZmNAC1 alleles* showed (1) differential expression in the parents (P-ratio≠1), (2) differential expression in the hybrid (H-ratio ≠1), or (3) a difference in the ratio of allelic expression in the parental lines relative to that of the hybrid (P-ratio ≠ H-ratio). Our analysis showed that the H-Ratio was 1.15 ± 0.06 (not significantly different from 1.0) (Table 1), indicating that there was no difference in cis-regulatory elements between alleles from 87-1 and Zong3. The P-Ratio (87-1/Zong3) was 0.58±0.01, which is significantly different from 1.0 (P < 0.05) and unequal to the H Ratio (P ratio≠H ratio) (Table 1), indicating that there was a change only in the trans-regulatory elements. These findings suggested that the trans-regulatory elements were the dominant mediator of the differential expression of *ZmNAC1* between the two Zong3 and 87-1.

**Table 1:** **Allele-specific transcript ratio of *ZmNAC1 *in hybrids and parents (cDNA-RT PCR)**

**87-1**	**Zong3**	**P Allelic ratio 87-1: Zong3**	**Zong3/87-1**		**H Allelic ratio 87-1: Zong3**
			**87-1 allele**	**Zong3allele**	
1.27(0.00)	2.21(0.05)	0.58(0.01)*	1.69(0.03)	1.48(0.1)	1.15(0.06)

The allelic transcript ratios (87-1: Zong3) of parents and hybrids were compared with an expected ratio indicating an equal allelic expression of 1.0. The mean, standard error (in parenthesis), and P values in the *t* test were based on three biological replicates. * P < 0.05.

**Table 2:** **Allele-specific transcript ratio of *Zm-miR164b *in hybrids and parents (cDNA-RT PCR)**

**87-1**	**Zong3**	**P Allelic ratio 87-1: Zong3**	**Zong3/87-1**		**H Allelic ratio 87-1: Zong3**
			**87-1 allele**	**Zong3 allele**	
4.41(0.02)	1.85(0.03)	2.39(0.03)* *	3.30(0.27)	1.48(0.17)	2.26(0.45) **

The allelic transcript ratio (87-1: Zong3) was significantly different from 1.0, with ** P < 0.01, whereas the P and H ratios were not significantly different.

### miR164 as the trans-acting factor that regulates expression of *ZmNAC1*

**Figure 5:**
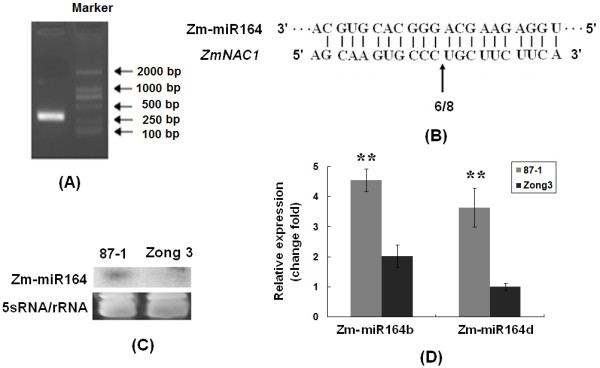
**Differential expression of *Zm-miR164 *between 87-1 and Zong3.A**. miR164 cleavage sites in *ZmNAC1* mRNA were determined by RNA ligase-mediated 5^′^ RACE. The resulting agarose gel showed the nested PCR products that we cloned and sequenced, and the product was expected to be approximately 300 bp. **B**. The frequency of 5’ RACE clones corresponding to the cleavage site (indicated by arrows) is shown as a fraction, with the number of clones matching the target message in the denominator. **C**. RNA gel blot analysis of *Zm-miR164* in 10 μg of low-molecular-weight RNA that was prepared from 8-day-old root samples from two separate inbred lines. A 5S rRNA sample was used as a loading control (bottom gels). **D**. Expression analysis of *miR164b* and *miR164d* with real-time quantitative reverse transcription-polymerase chain reaction (RT-PCR). Eight-day-old root seedling samples were used for the RNA extraction.

We assessed whether maize miR164 is the trans-acting factor that can direct the cleavage of *ZmNAC1*, and demonstrated that miR164 can direct *ZmNAC1* mRNA cleavage, by using a modified RNA ligase-mediated 5' rapid amplification of cDNA ends (5'-RACE) protocol. RNA sequences with 5' termini corresponding to the center of the miR164 complementary site were consistently detected as the product of miRNA processing. Sequencing analysis of 8 independent clones produced identical results and placed the 5' end of the cleaved fragment at nucleotide 991 of the *ZmNAC1* mRNA. This nucleotide position was located in the middle of the miR164/*NAC* mRNA complementary region, indicating that *ZmNAC1* mRNA was the in vivo miR164 cleavage target (Figure 5A and B).

We then investigated the expression of mature miR164 to determine whether miR164, which can act as the trans-acting regulator of *ZmNAC1*, also showed differential expression between 87-1 and Zong3. Northern blot analysis showed that the mature miR164 had higher expression levels in 87-1 than in Zong3 (Figure 5C), which was the opposite of the *ZmNAC1* expression pattern in the two inbred lines.

MiR164 was potentially transcribed from 8 loci, specifically from miR164a to miR164h, in maize. The mature miR164 from these loci differed by one or two nucleotides at the 3' end. We designed specific primers to amplify the 8 precursors, and only miR164b and miR164d had a higher expression level in the roots than in other tissues. Because the oligonucleotide probe used in the RNA gel blot cannot discriminate among these eight transcripts, we used gene-specific RT-PCR to analyze pri-miR164 expression. The results revealed that *pri-miR164b* (primary miRNA) and *pri-miR164d* showed 2.4-fold and 3.6-fold higher expression levels in 87-1 than in Zong3 (P < 0.01), respectively (Figure 5D), suggesting that a higher expression of miR164 precursors may contribute to the higher expression of mature miR164s in 87-1.

### *ZmmiR164b* allelic expression between maize hybrids and parents

**Figure 6:**
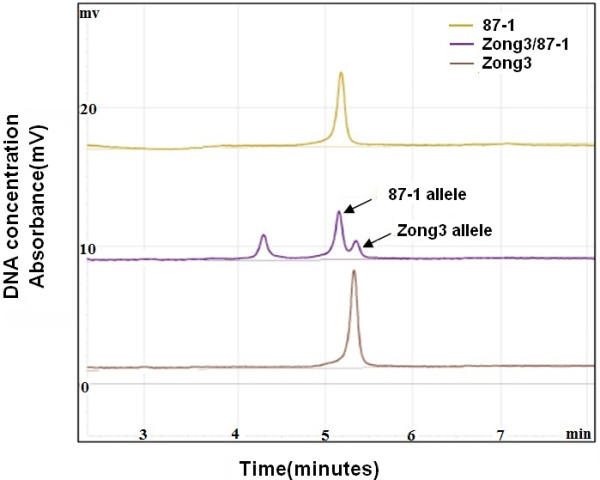
**RT-PCR and allele-specific cDNA quantification analysis of *ZmmiR164b *with the WAVE dHPLC system.***ZmmiR164b* allele from Zong3 contained 8-bp insertion as compared to that from 87-1. RT-PCR was then performed using primers designed for the conserved region between the alleles that encompassed the 8 bp Indel region. The RT-PCR products were separated and quantified using a WAVE dHPLC system. The longer DNA fragments that corresponded to the Zong3 allele had a higher affinity and therefore took a longer time to be eluted from the WAVE column than the shorter DNA fragments of the 87-1 allele eluted earlier. The x-axis shows the time in minutes when the DNA fragments were eluted. The y-axis demonstrates the UV absorbance that was used to measure DNA concentration or expression level. This analysis quantifies the allele-specific transcript as a relative ratio and does not measure the absolute transcription level.

The WAVE HPLC system was used to determine whether the differential expression of *ZmmiR164* precursors was regulated by a cis-or trans-acting mechanism. First, the full-length cDNA of the *miR164b* and *miR164d* precursors was obtained by 5′RACE, and the subsequent sequence analysis indicated that the single transcription start sites for *pri-miR164b* and *pri-miR164d* were 105 and 126 nucleotides upstream from the start of the mature miR164, respectively. Based on the full-length cDNA, the alleles from Zong3 and 87-1 were amplified and sequenced. The *ZmmiR164b* allele from Zong3 contained an 8 bp insertion in comparison to that of 87-1; this insert did not have an effect on the formation of the pre-miR164 secondary structure (Additional file 6). The allelic transcript ratio between the two parents (P value) and in the hybrid (H value) was determined by HPLC (Figure 6), and the results showed that the P ratio (87-1/Zong3) and H ratio (87-1/Zong3) could reach up to 2.39 ± 0.03 and 2.26 ± 0.45, respectively (Table 2). The P ratio = H ratio ≠ 1 pattern indicated that a cis-element was involved in the different expression patterns of *pri-miR164b* transcripts in the two inbred lines. Therefore, the sequence difference between the promoters of *ZmmiR164b* in Zong3 and 87-1 could be responsible for the differential expression of *ZmmiR164b*. For *ZmmiR164d*, the two alleles from the two inbred lines only differed at one nucleotide, so we could not detect the allelic expression with the WAVE dHPLC system.

### Promoters of *Zm-miR164b* from inbred line 87-1 showed higher activity

To provide evidence for the cis-regulation of the miR164 precursor, we isolated a 2.6 kb region upstream of the *miR164b* transcription start site and then determined the activity of the promoters from the two inbred lines 87-1 and Zong3. Sequence analysis revealed that the promoters from these two lines exhibited 71.07% nucleotide identity for *miR164b*. Based on the sequence polymorphism, specific primers were designed to differentiate the promoters from the two inbred lines.

**Figure 7:**
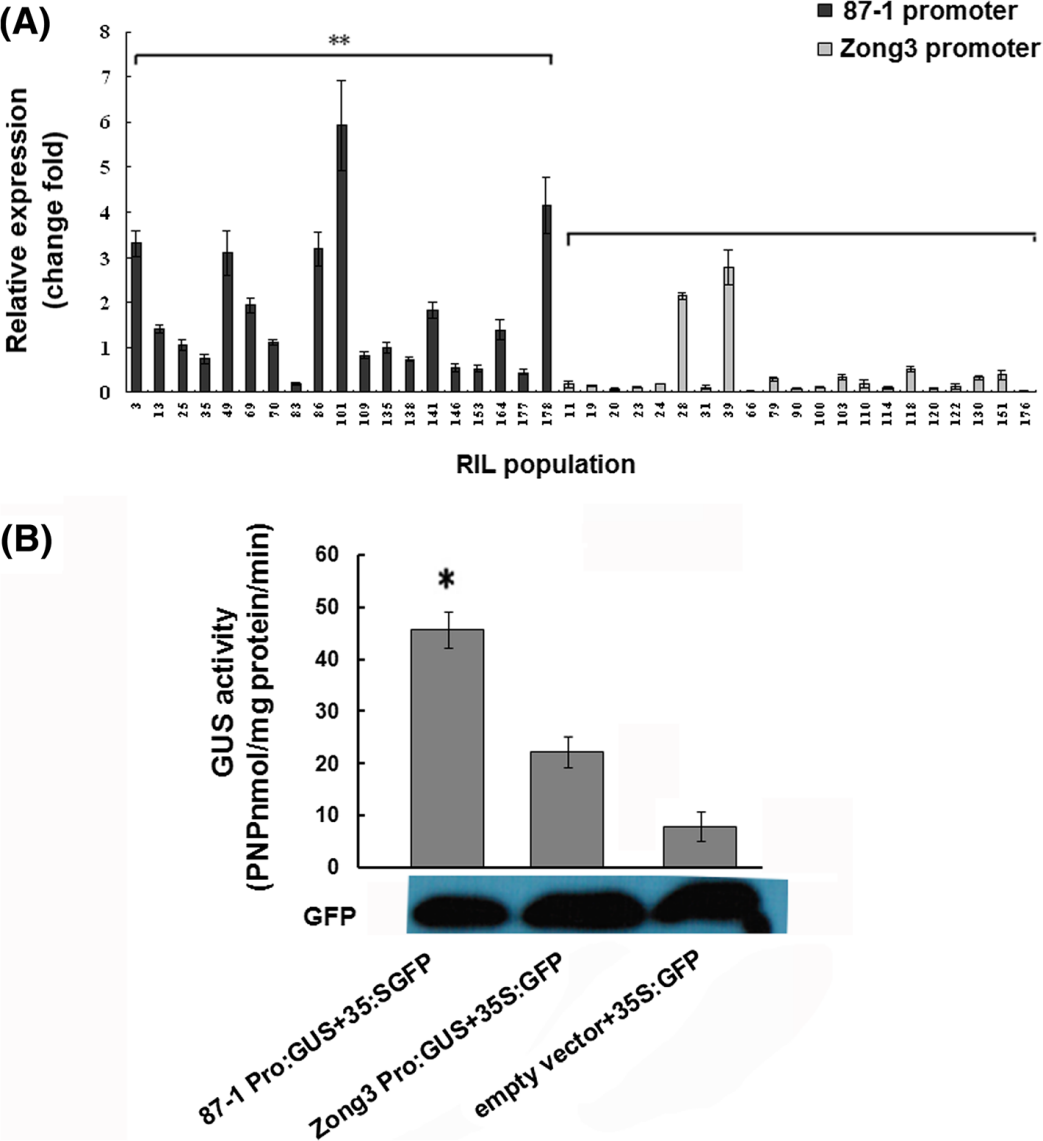
**Analysis of *pri-miR164b *promoter activity.A**. Expression analysis of *Zm-miR164b* in the RIL population. The darker columns were the RIL lines that contained the same promoter with 87-1, while the lighter ones had the same promoter with Zong3. ** represented significant difference between RIL plants containing the promoter from 87-1 and RIL plants containing the Zong3 promoter. **B**. GUS activity (nmole PNP/mg protein/min) as analysis driven by different promoters. GUS activity that was driven by the *Zm-miR164b* promoter from 87-1 and Zong3 in the tobacco leaf tissues was expressed as p-nitrophenol (PNP) nmole mg^-1^ protein min^-1^. The averages of the GUS activity and the standard deviations of the experiment were derived from three independent assays of tobacco leaf extracts. The significance of GUS activity differences relative to empty vector values as determined by Student’s *t*-test analysis is indicated here with asterisks (∗P < 0.05).

First, we tested whether *ZmmiR164b* showed higher transcription levels in RIL plants that contained the promoter from 87-1 than RIL plants that contained the Zong3 promoter. The promoter polymorphism was detected in Zong3, 87-1 and their 40 RILs, among which 19 lines had the same promoter as 87-1 and 21 lines had the same promoter as Zong3. We further examined the expression level of *ZmmiR164b* among these lines and found that the RILs with the 87-1 promoter had a higher expression level, with 4.37-fold (P < 0.01) on average compared with the lines with Zong3 promoters (Figure 7A). These findings strongly suggest that the cis-element of the miR164 promoter is the main contributor to the differential expression of *pri-miR164,* and it could be argued that other elements might also be involved in the regulation of *pri-miR164* expression*.*

To further investigate whether the *ZmmiR164b* promoter from 87-1 showed higher activity *in vitro* than that from Zong3, we designed a promoter: GUS reporter assay, including 87p-*ZmmiR164b*: GUS (the promoter of *miR164b* from 87-1) and Zong3p-*ZmmiR164b*: GUS (the promoter of *miR164b* from Zong3). Tobacco leaves were infiltrated with Agrobacterium that harbored these constructs, and GUS activity expression was analyzed by spectrophotometric assays. We also transformed tobacco leaves with a GFP vector to normalize the transformation efficiency. Our results showed that GUS activity that was driven by the promoter from 87-1was significantly higher than that driven by the promoter from Zong3 and by the background (Figure 7B), which strongly suggested that the different promoter activities of *pri-miR164* resulted in the diversity of *pri-mir164* transcript levels that was found between the two inbred lines, which may have led to the difference in mature miR164 expression between 87-1 and Zong3. In addition, the correlation coefficient between the expression levels of *ZmmiR164b* and the lateral root numbers among the RILs was also calculated, but no significant correlation was observed (Additional file 7).

## Discussion

### *ZmNAC1* played an important role in lateral root development

We isolated and characterized *ZmNAC1*, which is a member of the NAC-domain gene family and the first NAC gene shown to be involved in maize lateral root development. We demonstrated that *ZmNAC1*has high amino acid similarity to a homologous gene, *NAC1*, in *Arabidopsis*, which has also been reported to play an important role in lateral root development. In contrast to the other members of its family, *ZmNAC1* showed higher expression levels in the roots than in other tissues. Moreover, *ZmNAC1* was more highly expressed in the roots of inbred Zong3 than inbred 87-1, and inbred Zong3 had more lateral roots than inbred 87-1. The RIL populations derived from the Zong3/87-1 hybrid showed significant differences in lateral root numbers and in *ZmNAC1* expression. There was a significant correlation (correlation coefficient value reached 0.41, P < 0.01) between the expression levels of *ZmNAC1* and the lateral root number phenotype of the RILs, illustrating that *ZmNAC1* is important for maize lateral root development. Overexpression of *ZmNAC1* in *Arabidopsis* resulted in a higher number of lateral roots than in the wild type, whereas no other dramatic phenotype change was detected in transgenic plants. However, once the lateral roots initiated in the transgenic plants, no further alterations in root development occurred. These results suggest that *ZmNAC1* is specifically involved in the control of lateral root initiation. The phenotype of transgenic plants that overexpress *ZmNAC1* was consistent with *NAC1* having its main function in the roots, as only lateral root development was affected.

Nonetheless, we cannot exclude the possibility that *ZmNAC1* plays roles in other developmental pathways. *ZmNAC1* also showed a higher expression level in the ears, stems and male spikes than in the leaves, so we speculated that *ZmNAC1* might also be involved in the branching development of ears and male spikes.

### miR164-mediated *ZmNAC1* mRNA cleavage in vivo

Plant miRNAs have been implicated in the control of various developmental processes, including leaf development [30-32], flower development [33] and lateral root development [18]. In *Arabidopsis*, miR164 has been predicted to target 5 NAC-domain transcripts: *NAC1, CUC1, CUC2, At5g07680* and *At5g61430*[17]. Among these targets, *CUC1* and *CUC2* contribute to embryonic and floral development, and *NAC1* plays important roles in lateral root development. Here we provide evidence showing that miR164 directs *ZmNAC1* mRNA cleavage in vivo at the 11^th^ nucleotide of the complementary miR164 binding site, and this cleavage was conserved between maize and *Arabidopsis*. In addition, *ZmmiR164* showed higher expression levels in inbred 87-1 than in Zong3, which was the opposite of the expression pattern found for *ZmNAC1*, suggesting that miR164 negatively regulates *ZmNAC1*. These findings confirmed that this conserved miRNA can regulate a target gene in a conserved pathway throughout evolution.

### miR164 as a trans-acting factor contributed to the differential expression of *ZmNAC1* between 87-1 and Zong3

The results of the allelic variation analysis for *ZmNAC1* in 87-1, Zong3, and hybrid Zong3/87-1 maize showed that the trans-acting factor, and not the cis-element, was most likely the major factor underlying the differential expression of *ZmNAC1* between 87-1 and Zong3. We further isolated and compared the 1 kb *ZmNAC1* promoter sequences between the inbred lines 87-1 and Zong3, and no sequence difference was found except for one SNP (Additional file 8). Based on the *ZmNAC1* mRNA cleavage that was directed by miR164, we consider miR164 to be one of the post-transcriptional trans-acting factors that regulates *ZmNAC1* expression. However, no significant negative correlation was detected between the expression levels of *ZmmiR164b* and *ZmNAC1* in the RIL population, suggesting that the expression of *ZmNAC1* is also regulated by other genes, as observed in *Arabidopsis*. For example, Xie reported that the F-box protein *TIR1* is likely involved in ubiquitin-mediated proteolysis of regulatory proteins that are required for an auxin response and can induce the expression of *NAC1* through auxin signals [8].

Further study revealed that both the mature miR164 and the precursor *pri-miR164b* were expressed at higher levels in 87-1 than in Zong3 and that the promoter of *ZmmiR164b* from 87-1 showed higher activity in vivo and *in vitro* than that of Zong3, which might lead to the higher expression level of miR164 in 87-1 than in Zong3. It has been reported that *Arabidopsis* miR164 negatively regulates lateral root development, showing a strict inverse correlation between changes in the miR164 level and the *NAC1* mRNA levels. Mutant plants that were defective in miRNA biogenesis showed a higher level of *NAC1* mRNA and more lateral roots. Conditional overexpression of miRNA164 decreased *NAC1* mRNA and lateral root numbers. Based on our results, it is possible to improve the root development in maize by altering the miR164 pathway.

*Arabidopsis NAC1* has been shown to be a transcriptional activator in auxin-induced lateral root initiation. This study shows that *ZmNAC1* also plays an important role in maize lateral root development and that *ZmNAC1* expression is regulated at the post-transcriptional level by miR164. Overall, our data suggest that in 87-1 maize (which has fewer lateral roots than Zong3), the *miR164b* promoter has higher activity than in Zong3, leading to a higher expression level of mature miR164, which then downregulates *ZmNAC1* expression at the post-transcriptional level. This pathway might contribute to the smaller lateral root numbers in 87-1. By contrast, both *pri-miR164b* and mature miR164 had a lower expression level in inbred Zong3 maize than in 87-1 maize, leading to a higher expression of *ZmNAC1,* thus contributing to greater numbers of lateral roots. It should be noted that no significant negative correlation was detected between the expression levels of *ZmmiR164b* and lateral root numbers in the RIL population, which revealed the complexity of *ZmmiR164* and its regulation of lateral root development in maize.

## Conclusion

*Arabidopsis NAC1* is a transcriptional activator in auxin-induced lateral root initiation. Our study shows that the maize homologue, *ZmNAC1*, also plays an important role in lateral root development in maize. Our study then extends the research by showing that *ZmNAC1* expression is regulated at the post-transcriptional level by maize miRNA164. Overall, our data suggest that the miR164b promoter showed higher activity in inbred 87-1 maize than in Zong3 maize, leading to higher expression of mature miR164, which down-regulated *ZmNAC1* expression at the post-transcriptional level. This pathway might contribute to 87-1 having fewer lateral root numbers than Zong3.

## Methods

### Plant materials and growth condition

The maize (*Zea mays* L) inbred lines 87-1 and Zong3, the hybrid line Zong3/87-1, and 40 recombinant inbred lines (RIL) that were derived from the hybrid Zong3/87-1 were used for this study. For phenotypic analysis of lateral root numbers, seed germination was initiated by soaking in distilled water in a Petri dish for 12h at room temperature. Seeds were then placed between layers of moist paper towels at 30°C for 24 h and transplanted into pots. Seeds were cultivated in vermiculite with no additional fertilizer and grown in a growth chamber at a relative humidity of 40-60% and a 26/24°C day and night temperature under a 16-h-light/8-h-dark photoperiod. Eight individuals were planted for each genotype, and half of them were collected for gene expression analysis (the rest were used for the investigation of lateral root density). All of the root samples were collected at 8 days after germination between 8:00 a.m. and 9:00 a.m., and root samples were collected carefully and cleaned with water without causing any injury. Lateral root numbers in primary root were counted manually, and the number was divided by the length of primary root to calculate the parameter for lateral root density [34]. Three biological replicates were performed for both gene expression and phenotypic analysis of lateral roots. For tissue expression pattern analysis, the root, leaf, sheath in the V3 stage, spike in the V12 stage, and stem in the V9 stage were collected for real-time PCR and Northern blot analysis.

The *Arabidopsis thaliana* Col-0 ecotype was used for transformation. Seeds were germinated after 3 d of vernalization on Murashige and Skoog (MS) medium at 4°C. Plants that were intended for transformation were grown under continuous light (150 mE/m^2^per s) at 22°C in a greenhouse.

### Gene expression analysis

For Northern blot hybridization, total RNA was isolated using Trizol (Invitrogen, Carlsbad, CA, USA) according to the manufacturer's instructions. Low molecular weight RNA was enriched with 0.5 M NaCl and 10% PEG 8000 precipitation. Ten μg of low molecular weight RNA was loaded per lane, resolved on a denaturing 15% polyacrylamide gel, and electrophoretically transferred to Hybond-N^+^ membranes (Amersham Biosciences, Buckinghamshire, UK). Membranes were UV-crosslinked and baked for 2 h at 80°C. DNA oligonucleotides that were complementary to miR164 were end-labeled with γ-32P-ATP using T4 polynucleotide kinase (TaKaRa, Dalian, China). Membranes were pre-hybridized for more than 8 h and then hybridized overnight using Church buffer at 38°C. Blots were washed three times (twice with 2 × SSC + 1% SDS and once with 1 × SSC + 0.5% SDS) at 50°C. The membranes were briefly air-dried and then exposed to X-ray films for autoradiography at −80°C.

For the real-time PCR analysis, total RNA was isolated by using Trizol (Invitrogen, USA) according to the manufacturer's instructions and treated with RNase-free DNase I (Promega, Madison, USA). Two μg of total RNA from each sample was used for first-strand cDNA synthesis in 20 μL reactions containing 50 mM Tris–HCl (pH 8.3), 75 mM KCl, 3 mM MgCl_2_, 10 mM DTT, 50 μM dNTPs, 200 U M-MLV reverse transcriptase (Promega, Madison, USA) and 50 pmol T15 oligonucleotides. Reverse transcription was performed at 37°C for 60 min with a final denaturation at 95°C for 5 min. Gene-specific RT-qPCR primers for 7 putative miR164 targets were designed according to the EST sequences. A 250 bp β-actin gene fragment was amplified as a positive control using the primer pair 5'-TGGCATTGTCAACAACTGG-3' and 5'-TCATTAGGTGGTCGGTGAGG-3'. PCR reactions were performed in a volume of 20 μL containing 10 pmol primers, 10 mmol/L Tris–HCl (pH8.5), 50 mmol/L KCl, 2 mmol/L MgCl_2_, 0.4 μL DMSO, 200 mmol/L dNTPs, 1 U Taq DNA Polymerase (TaKaRa, Dalian), and 0.5 μL SYBR GREEN I. The following PCR amplification protocol was used: 95°C (3 min) and 40 cycles of amplification cycle (95°C (30 s), 55°C (30 s),and 72°C (1 min)) using an Opticon PTC200 system (MJ Research, Waltham, MA, USA). All reactions were run in triplicate, and no template and no reverse transcription controls were included. Quantification results were expressed in terms of the cycle threshold (CT) values according to the baseline, which was adjusted to 0.04. The comparative CT method (PE Applied Biosystems, Foster City, CA, USA) was used to quantify gene expression in comparison with actin. In brief, the CT values were averaged for each triplicate. The differences between the mean CT value of a specific gene and that of β-actin was calculated as ΔCT ^sample^ = CT^gene^-CT^β-actin^. In the final results, the sample's relative expression level was determined by 2^-ΔCt^ method. Statistical significance was tested using Student’s *t*-test (P < 0.05).

### Mapping of the miRNA-guided cleavage site

The GeneRacer Kit (Invitrogen USA) was used for the RACE assay according to the manufacturer’s instructions. Total RNA was extracted from the roots of 87-1 seedlings, and Poly(A)mRNA was purified and ligated to the RLM-RACE 5'RACE RNA Oligo adaptor (5'-CGACUGGAGCACGAGGACACUGACAUGGACUGAAGGAGUAGAAA-3'). The oligo (dT) (15-mer) primer was used for cDNA synthesis with reverse transcriptase, and the resultant cDNA was used for the first round of nested PCR using the 5' RACE primer 5'-CGACTGGAGCACGAGGACACTGA-3' together with *ZmNAC1* gene-specific primer 5’-ACCCAAGCCTCTTGTAGCACTCATC-3'. The 5' RACE nested primer 5'-GGACACTGACATGGACTGAAGGAGAT-3' and the gene-specific nested primer 5'-GTCGAGGCATTTCGATCCGCATC-3' were used for the second round of nested PCR. Gel-purified PCR products were cloned into the pGEM-T Easy Vector (Promega) and sequenced.

### Transformation vectors and construction of transgenic plants

For the transgenic *Arabidopsis* plants, constructs were made in the binary vector pCAMBIASuper1300. For the plasmid super-p: *ZmNAC1*, the forward primer (5'-GCTCTAGACGCAGAAGTTGACCACGTAC-3') (the underlined sequence is the XbaI site) and the reverse primer (5'-GGGGTACCATCCATCCTGTTATCGTCGAG-3') (the underlined sequence is the KpnI site) were designed to introduce XbaI and KpnI sites, and Zong3 root cDNA samples were used to amplify a 1463 bp cDNA from *ZmNAC1*. The construct was transformed into Agrobacterium GV3101. Six-week-old *Arabidopsis* plants were transformed via Agrobacterium-mediated transformation by the floral-dip method [35]. Transgenic plants were selected on 1/2 MS medium (Gibco BRL, Grand Island, NY, USA) containing hygromycin B. T3 generation was used for further experiments.

### Agrobacterium-mediated transient expression assay and GUS activity assay

The miR164b promoters from the inbred lines 87-1 and Zong3 were ligated into the Gateway pDONR 211Vector (Invitrogen USA), and then the vector miR164b-87-1promoter:: GUS and miR164b-Zong3promoter::GUS construct made according to the manufacturer’s instructions. Two primers in different orientations (5'-CAAAAAAGCAGGCTGTGATTGACGACAACATGAACAAATC-3',5'-ACAAGAAAGCTGGGTCGCAATTCTCGAATTCACCTTC-3'; the underlined sequence is the attB1 and attB2 site) were used to amplify the promoter sequences from 87-1 and Zong3 in separate DNA samples. Agrobacterium GV3101 that harbored each of the promoter:GUS constructs was grown on yeast extract peptone medium (10 g yeast extract, 10 g Bacto peptone, 5 g NaCl, and 15 g agar/l) supplemented with rifampicin (60 μg/mL), kanamycin (50 μg/mL) and spectinomycin (100μg/mL). Agrobacterium was cultured at 28°C and harvested by centrifugation for 15 min at 6,000 g, re-suspended in infiltration media (10 mM MgCl_2_ and 10 mM MES containing 150 mM acetosyringone) for 2 h at room temperature to induce T-DNA transfer functions [36], and then adjusted to an OD600 of 0.8. After the infiltration of the Agrobacterium suspension into the abaxial surfaces of tobacco leaves from a syringe without a needle [37], the tobacco plants were maintained in a moist chamber at 26°C for 48 h for GUS activity analysis.

GUS activity in these tobacco leaves was measured as described by Jefferson [38]. Tobacco leaf tissues were homogenized in 1 ml of extraction buffer (50 mM NaH_2_PO_4_, pH 7.0, 10 mM EDTA, 0.1% Triton X-100, 0.1% (w/v) sodium laurylsarcosine, 10 mM β-mercaptoethanol, and 6 mM L-ascorbic acid). After centrifugation for 10 min at 12,000 rpm (4°C), the supernatant was transferred to a fresh microtube. The spectrophotometric reaction was carried out in a 1 mL volume with 1 mM PNPG (P-nitrophenyl-β-D-glucuronide) in the extraction buffer supplemented with protein extract supernatants. GUS activity was normalized to the protein concentration in each of the crude extracts and was expressed as nmol of P-nitrophenol min/mg protein. The total protein in the sample extract was quantified using bovine serum albumin as a standard, according to the Bradford method [39]. The GUS measurement was repeated at least three times.

### RT-PCR analysis using the WAVE dHPLC system

Total RNA was isolated using Trizol (Invitrogen, Carlsbad, CA, USA) according to the manufacturer's instructions. We used gene-specific primers (5'-CAGCTCCACACCTGTACGT-3' and 5'-CCATGCTCAGCGACTTGATG-3' for *ZmNAC1*, and 5'-ACGTGCATTACCATCCAATGC-3', 5'-CTGCATGACGAGGTATGTACG-3' for *ZmmiR164b*) to obtain the corresponding cDNA from each inbred line by RT-PCR. The PCR products were then sequenced to identify allele-specific sequence polymorphisms between the inbred lines 87-1 and Zong3 that would allow separation of the two parental alleles on the WAVE dHPLC system (Transgenomic, Omaha, NE). The gene-specific primers were designed to amplify the regions that are conserved between alleles and that encompass a sequence polymorphism indel to minimize the amplification preference of either allele and to optimize the amplicon for WAVE analysis. The β-actin gene fragment was amplified as a positive control using 26 cycles. Three PCR replicates were performed for each RNA sample. The RT-PCR products were then separated and quantified by the WAVE dHPLC system, and detailed WAVE dHPLC analysis was performed according to the manufacturer’s instructions [40].

## Authors’ contributions

Jing Li and Guanghui Guo carried out the main experiments and drafted the manuscript, and they contributed equally to this study. Weiwei Guo, Ganggang Guo, and Dan Tong greatly helped the first author in the transgenic work, RIL analysis and Northern blot analysis. Zhongfu Ni contributed by analyzing the data. Yingyin Yao and Qixin Sun designed the research and finished the manuscript. All authors read and approved the final manuscript.

## Supplementary Material

Additional file 1 **Polygenetic tree of 175 maize NAC and 105*Arabidopsis*NAC proteins.** The unrooted phylogenetic tree of NAC proteins was constructed using the CLUSTAL X program with the neighbor-joining method. In this figure, 105 Arabidopsis NAC proteins and 175 maize NAC proteins are classified into three large groups, separated by blue lines. Red arcs represent 14 subgroups and pink dots denote putative miR164 target genes in maize. The sequences of 105 Arabidopsis NAC proteins are from reference [3]. Click here for file

Additional file 2 **Putative ZmNACs were obtained as the putative miR164 target genes.** This figure shows the reverse complementary site for mature miR164 and 7 ZmNACs. Click here for file

Additional file 3 **The full-length cDNA of *ZmNAC1.*** This figure shows the full length cDNA of *ZmNAC1*, in which the highly conserved region of the NAC domain is boxed; the red box indicates the (NLS) nuclear localization signal as noted between amino acids 121 and 138. Click here for file

Additional file 4 **The figure shows the variation of lateral root density among RILs.** 40 RILs were investigated 8days after germination, 4 plants per genotype were investigated for lateral root density. Lateral root numbers in primary root were counted manually and was divided by the total length of the primary root as a parameter for lateral root density (De Smet et al. 2012). Analysis of variance for the lateral root density among 40 RILs were performed. Click here for file

Additional file 5 **The expression levels of*ZmNAC1*among RILs.** This figure shows the expression of *ZmNAC1* in the RILs, which was investigated 8 days after germination by planting eight plants for each genotype, half of which were collected for gene expression analysis. Click here for file

Additional file 6 **The secondary structure of ZmmiR164b in 87-1 and Zong3.** This figure shows the secondary structure of ZmmiR164b in 87-1 and Zong3 as determined by mFold software. The good hairpin structure indicates that both of them can give rise to mature miR164. Click here for file

Additional file 7 **The correlation between lateral root numbers and expression of mir164b in 40 RILs.** This figure shows that a large variation exists in both lateral root numbers and the expression of miR164b among 40 RILs. The correlation coefficient value was calculated, and no significant correlation was found. Click here for file

Additional file 8 **Comparison of the*ZmNAC1*promoter between 87-1 and Zong3.** This figure shows that no large variation exists in the 1 kB region of the *ZmNAC1* promoter between two inbred lines, with the exception of one SNP. Click here for file
