# Supplementary material for: miRNA164-directed cleavage of ZmNAC1 confers lateral root development in maize (Zea mays L.)
Source: BMC Plant Biol. 2012 Nov 21;12:220. doi: 10.1186/1471-2229-12-220 (PMC3554535; doi:10.1186/1471-2229-12-220)
Supplement: Additional file 1 — Polygenetic tree of 175 maize NAC and 105ArabidopsisNAC proteins. The unrooted phylogenetic tree of NAC proteins was constructed using the CLUSTAL X program with the neighbor-joining method. In this figure, 105 Arabidopsis NAC proteins and 175 maize NAC proteins are classified into three large groups, separated by blue lines. Red arcs represent 14 subgroups and pink dots denote putative miR164 target genes in maize. The sequences of 105 Arabidopsis NAC proteins are from reference [3]. [file 1471-2229-12-220-S1.pdf]

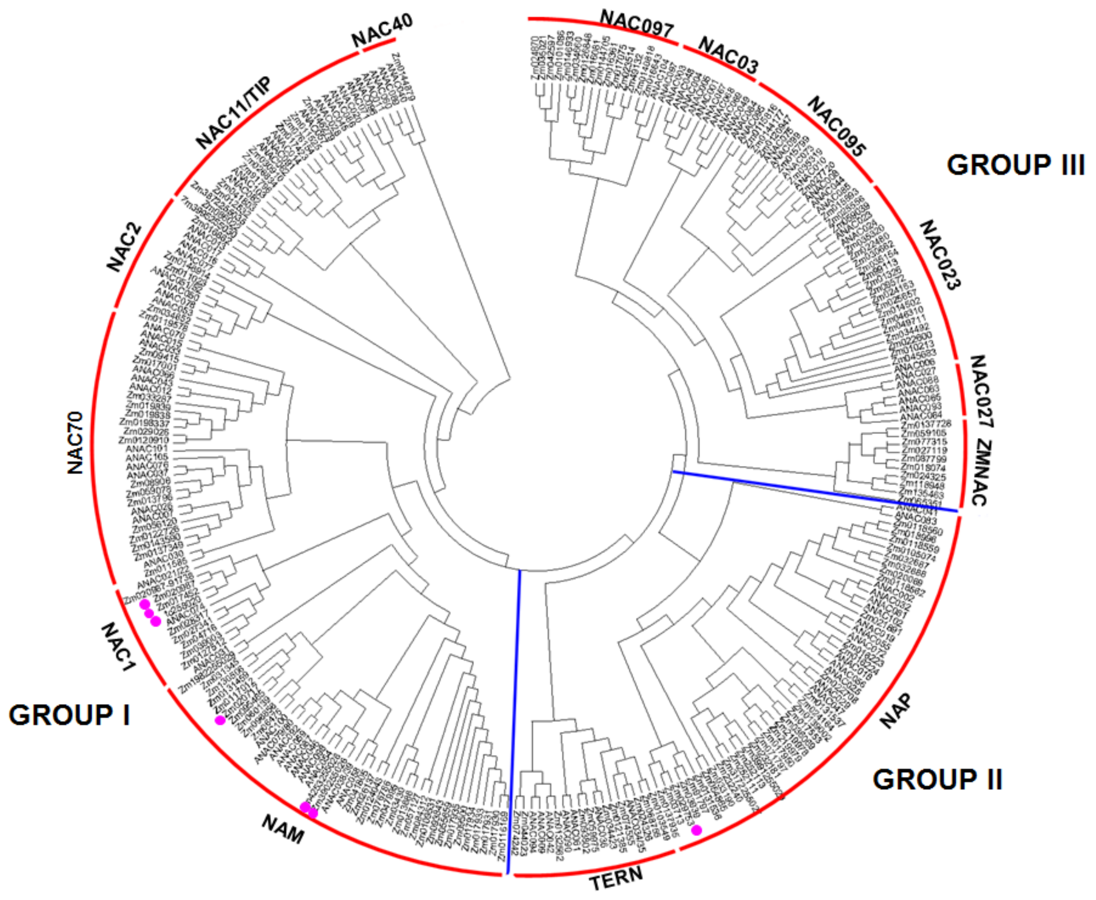

Additional file 1. Polygenetic tree of 175 maize NAC and 105 *Arabidopsis* NAC proteins

The unrooted phylogenetic tree of NAC proteins was constructed using the CLUSTAL X program with the neighbor-joining method. In this figure, 105 Arabidopsis NAC proteins and 175 maize NAC proteins are classified into three large groups, separated by blue lines. Red arcs represent 14 subgroups and pink dots denote putative miR164 target genes in maize. The sequences of 105 Arabidopsis NAC proteins are from reference [3].
