# Supplementary material for: miRNA164-directed cleavage of ZmNAC1 confers lateral root development in maize (Zea mays L.)
Source: BMC Plant Biol. 2012 Nov 21;12:220. doi: 10.1186/1471-2229-12-220 (PMC3554535; doi:10.1186/1471-2229-12-220)
Supplement: Additional file 2 — Putative ZmNACs were obtained as the putative miR164 target genes. This figure shows the reverse complementary site for mature miR164 and 7 ZmNACs. [file 1471-2229-12-220-S2.pdf]

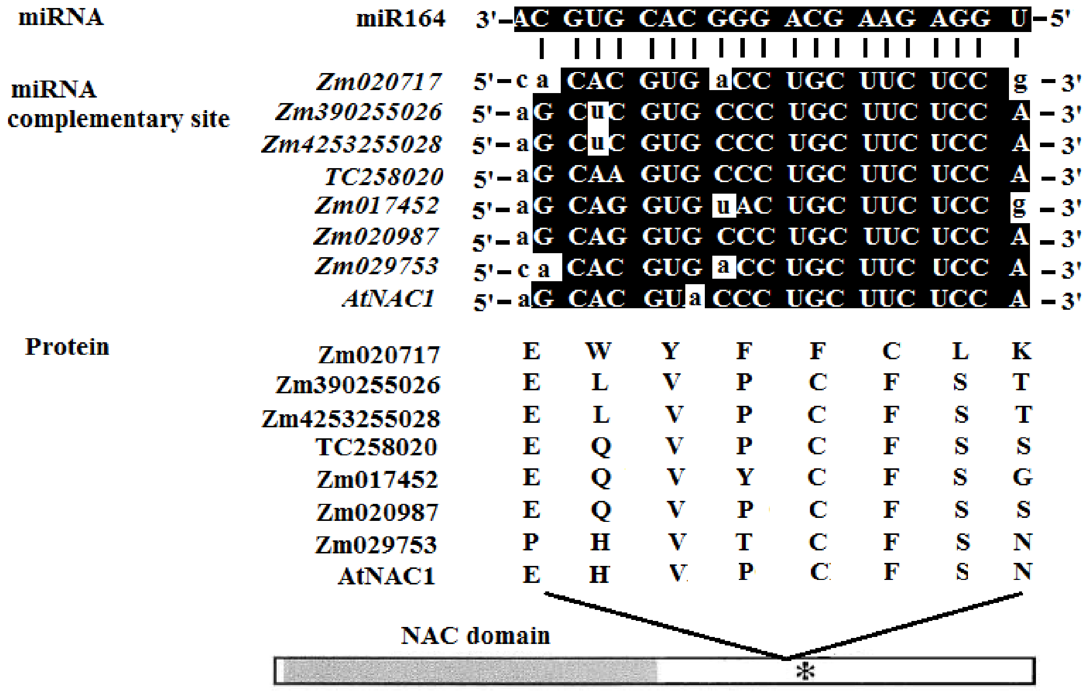

Additional file 2. Putative ZmNACs were obtained as the putative miR164 target genes

This figure shows the reverse complementary site for mature miR164 and 7 ZmNACs.
