# Supplementary material for: miRNA164-directed cleavage of ZmNAC1 confers lateral root development in maize (Zea mays L.)
Source: BMC Plant Biol. 2012 Nov 21;12:220. doi: 10.1186/1471-2229-12-220 (PMC3554535; doi:10.1186/1471-2229-12-220)
Supplement: Additional file 3 — The full-length cDNA of ZmNAC1. This figure shows the full length cDNA of ZmNAC1, in which the highly conserved region of the NAC domain is boxed; the red box indicates the (NLS) nuclear localization signal as noted between amino acids 121 and 138. [file 1471-2229-12-220-S3.pdf]

```
1      AAAAACCCAAGAAAAGTCTCTCTCTTTCTCTCTCTCCTAGCTAGCCCCCGATCTGGCCCT

61     CTCTCTCACGCGCTCTAGAACTAGAAGGGCATCCGCAGAAGTTGACCACGTACAGCAGCG

121    GCAGCGCGGGCTCCTCCTGTTATATAACGCCCGCCTCTCGCCGCTCTCCAGCTTCTTCTT

181    AATTTCTCTTCTATGATACAGGGGTAGGAGAAGGAGGAGGAGGAGTAGAAGCAGCTCCAC

241    ACCTGTACGTAGCTGCCGCGCGCCGGTGAGCGAGGTCGTTCTTGAGAGCTGTATATATAT

301    ATATAGCTCCGCGACGACGCCGATGATGGCCATCAAGTCGCTGAGCATGGTGGAGGCCAG
1                              M  M  A  I  K  S  L  S  M  V  E  A  S

361    CCTGCCTCCGGGGTTCAGGTTCCACCCGCGCGACGACGAGCTCGTGCTGGACTACCTGGC
14      L  P  P  G  F  R  F  H  P  R  D  D  E  L  V  L  D  Y  L  A

421    CAAGAAGCTCGGCGGCGGCGGAGGCCCTGTGGTGGTGAGCATCTACGGCTGCCCCACCAT
34      K  K  L  G  G  G  G  G  P  V  V  V  S  I  Y  G  C  P  T  M

481    GGTCGACGTCGATCTCAACAAGTGCGAGCCCTGGGACCTTCCCGACATCGCGTGCATTGG
54      V  D  V  D  L  N  K  C  E  P  W  D  L  P  D  I  A  C  I  G

541    TGGAAAAGAGTGGTATTTCTACAGCCTTAGAGATAGAAAGTATGCCACTGGCCAGCGTAC
74      G  K  E  W  Y  F  Y  S  L  R  D  R  K  Y  A  T  G  Q  R  T

601    AAACAGAGCAACTGATTCGGGATACTGGAAGGCCACAGGGAAAGACCGTCCAATAAGCCG
94      N  R  A  T  D  S  G  Y  W  K  A  T  G  K  D  R  P  I  S  R

661    GAAAGGGTTACTTGTTGGTATGCGCAAAACCCTTGTGTTTTATCAAGGTAGAGCCCCAAA
114     K  G  L  L  V  G  M  R  K  T  L  V  F  Y  Q  G  R  A  P  K

721    GGGAAAGAAGACCGAGTGGGTTATGCATGAGTTTCGCATGGAAGGGCGAGATGATCCCAT
134     G  K  K  T  E  W  V  M  H  E  F  R  M  E  G  R  D  D  P  M

781    GAAATTACCTTTCAAGGAGGACTGGGTCTTGTGTAGAGTTTTCTACAAGAGTAGGGCAAC
154     K  L  P  F  K  E  D  W  V  L  C  R  V  F  Y  K  S  R  A  T

841    AGTTGCAAAGCCGCCCACAGAGAGCAGCAGCTTCAATATTGATGCAGCCACAACTTCATT
174     V  A  K  P  P  T  E  S  S  S  F  N  I  D  A  A  T  T  S  L

901    GCCTCCCCTTATTGACAACAACTTCAATATCTCCTTTGACCAGCCTGGCTCATCATCAGT
194     P  P  L  I  D  N  N  F  N  I  S  F  D  Q  P  G  S  S  S  V

961    GCAGAACCTAGAGGGTTATGAGCAAGTGCCCTGCTTCTCCAGTAACCCCTCTCAGCAGCC
214     Q  N  L  E  G  Y  E  Q  V  P  C  F  S  S  N  P  S  Q  Q  P

1021   ATCGTCGTCGATGAACGCCGCCCGGCTGCCGCCGTCTGCCGCCATGGCTGATCCGGAGCA
234     S  S  S  M  N  A  A  R  L  P  P  S  A  A  M  A  D  P  E  Q

1081   GCAGATGGGGAAGTCAATAATCAAGGATGTTCTCATGAGCCAGTTTAGCAGGTTCGAAGG
254     Q  M  G  K  S  I  I  K  D  V  L  M  S  Q  F  S  R  F  E  G

1141   CAGCGTCAAGAGGGAGGCGCCTCCAAGCAATTTTTCTCAGGATGGGTTTGAGTACTTAGC
274     S  V  K  R  E  A  P  P  S  N  F  S  Q  D  G  F  E  Y  L  A

1201   TGAGAGTGGCTTCACGCAGATGTGGAATTCGTTCAATTAGTTGATTATGTTTTATAGGAA
294     E  S  G  F  T  Q  M  W  N  S  F  N  *

1261   GATTATATATATATTATTGTTATGGAGAGCGATGCGGATCGAAATGCCTCGACGATAACA

1321   GGATGGATATATAGATGAGTGCTACAAGAGGCTTGGGTGTGTGTGCCTCCTGTGTGGTAT

1381   TGCATGTAGCCGTAGTAGTAGTAGGTATATATATACATATAGTAGCAGTACCATCTCAGT

1441   ACACATCCTTCAAAAAGAAGGAGAAATGAAAAGAGAGATATATATATGTCTGTATGCCCT

1501   ATGCCATCAATTAATGACGACAGATGTGTAAATACATTATTTGTGTACTATGGAGAAATC

1561   AGAAGGAAAAAAATACAGTGCGTACGTGATGACAAAAAACAAGAATAGAATTTTATTCAT

1621   ATGGTCCGTTGTCTATTAAAAAAAAAAAAAAAAAAAA
```

Additional file 3. The full-length cDNA of *ZmNAC1*

This figure shows the full length cDNA of *ZmNAC1*, in which the highly conserved region of the NAC domain is boxed; the red box indicates the (NLS) nuclear localization signal as noted between amino acids 121 and 138.
