# Supplementary material for: miRNA164-directed cleavage of ZmNAC1 confers lateral root development in maize (Zea mays L.)
Source: BMC Plant Biol. 2012 Nov 21;12:220. doi: 10.1186/1471-2229-12-220 (PMC3554535; doi:10.1186/1471-2229-12-220)
Supplement: Additional file 4 — The figure shows the variation of lateral root density among RILs. 40 RILs were investigated 8days after germination, 4 plants per genotype were investigated for lateral root density. Lateral root numbers in primary root were counted manually and was divided by the total length of the primary root as a parameter for lateral root density (De Smet et al. 2012). Analysis of variance for the lateral root density among 40 RILs were performed. [file 1471-2229-12-220-S4.pdf]

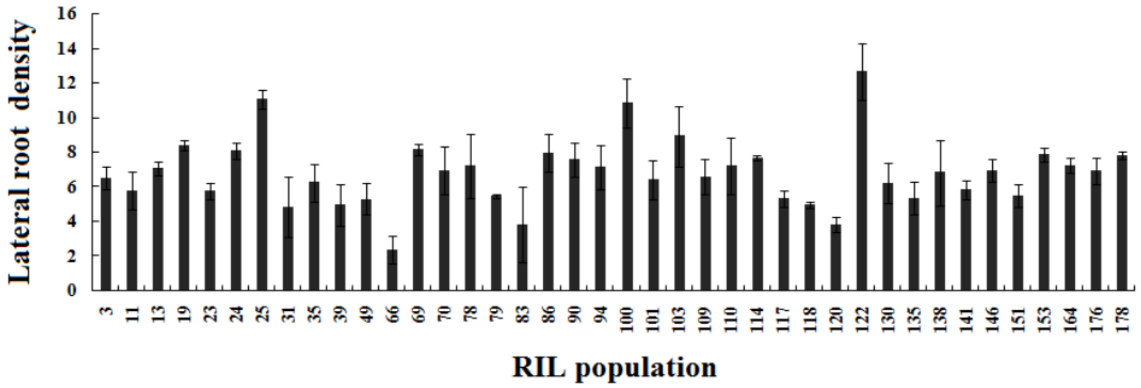

Analysis of Variance

Different source	SS	df	MS	F	P-value	F crit
Genotypes	441.74	39	11.33	7.95	3.84E-15	1.55
Errors	113.94	120	1.42			
Total	555.68	159				

Additional file 4. The investigation of lateral root numbers among RILs

The figure shows the variation of lateral root density among RILs. 40 RILs were investigated 8days after germination, 4 plants per genotype were investigated for lateral root density. Lateral root numbers in primary root were counted manually and was divided by the total length of the primary root as a parameter for lateral root density (De Smet et al. 2012). Analysis of variance for the lateral root density among 40 RILs were performed .
