# Supplementary material for: miRNA164-directed cleavage of ZmNAC1 confers lateral root development in maize (Zea mays L.)
Source: BMC Plant Biol. 2012 Nov 21;12:220. doi: 10.1186/1471-2229-12-220 (PMC3554535; doi:10.1186/1471-2229-12-220)
Supplement: Additional file 5 — The expression levels ofZmNAC1among RILs. This figure shows the expression of ZmNAC1 in the RILs, which was investigated 8 days after germination by planting eight plants for each genotype, half of which were collected for gene expression analysis. [file 1471-2229-12-220-S5.pdf]

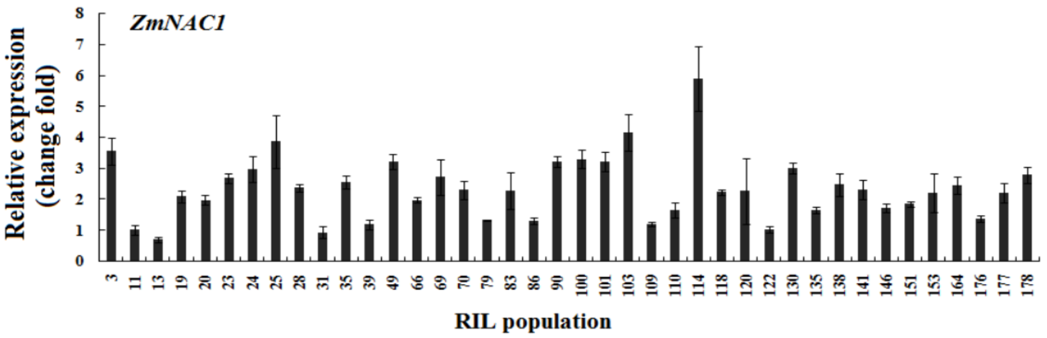

Additional file 5. The expression levels of *ZmNAC1* among RILs

This figure shows the expression of *ZmNAC1* in the RILs, which was investigated 8 days after germination by planting eight plants for each genotype, half of which were collected for gene expression analysis.
