# Supplementary material for: miRNA164-directed cleavage of ZmNAC1 confers lateral root development in maize (Zea mays L.)
Source: BMC Plant Biol. 2012 Nov 21;12:220. doi: 10.1186/1471-2229-12-220 (PMC3554535; doi:10.1186/1471-2229-12-220)
Supplement: Additional file 6 — The secondary structure of ZmmiR164b in 87-1 and Zong3. This figure shows the secondary structure of ZmmiR164b in 87-1 and Zong3 as determined by mFold software. The good hairpin structure indicates that both of them can give rise to mature miR164. [file 1471-2229-12-220-S6.pdf]

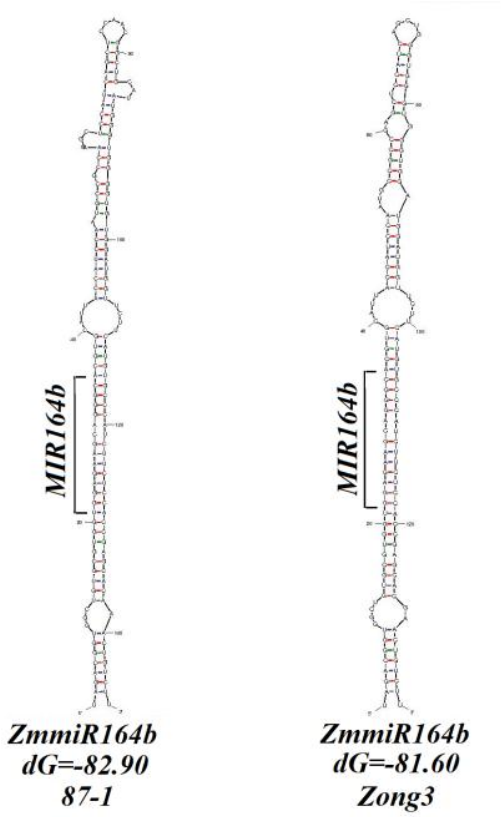

Additional file 6. The secondary structure of ZmmiR164b in 87-1 and Zong3

This figure shows the secondary structure of ZmmiR164b in 87-1 and Zong3 as determined by mFold software. The good hairpin structure indicates that both of them can give rise to mature miR164.
