# Supplementary material for: miRNA164-directed cleavage of ZmNAC1 confers lateral root development in maize (Zea mays L.)
Source: BMC Plant Biol. 2012 Nov 21;12:220. doi: 10.1186/1471-2229-12-220 (PMC3554535; doi:10.1186/1471-2229-12-220)
Supplement: Additional file 7 — The correlation between lateral root numbers and expression of mir164b in 40 RILs. This figure shows that a large variation exists in both lateral root numbers and the expression of miR164b among 40 RILs. The correlation coefficient value was calculated, and no significant correlation was found. [file 1471-2229-12-220-S7.pdf]

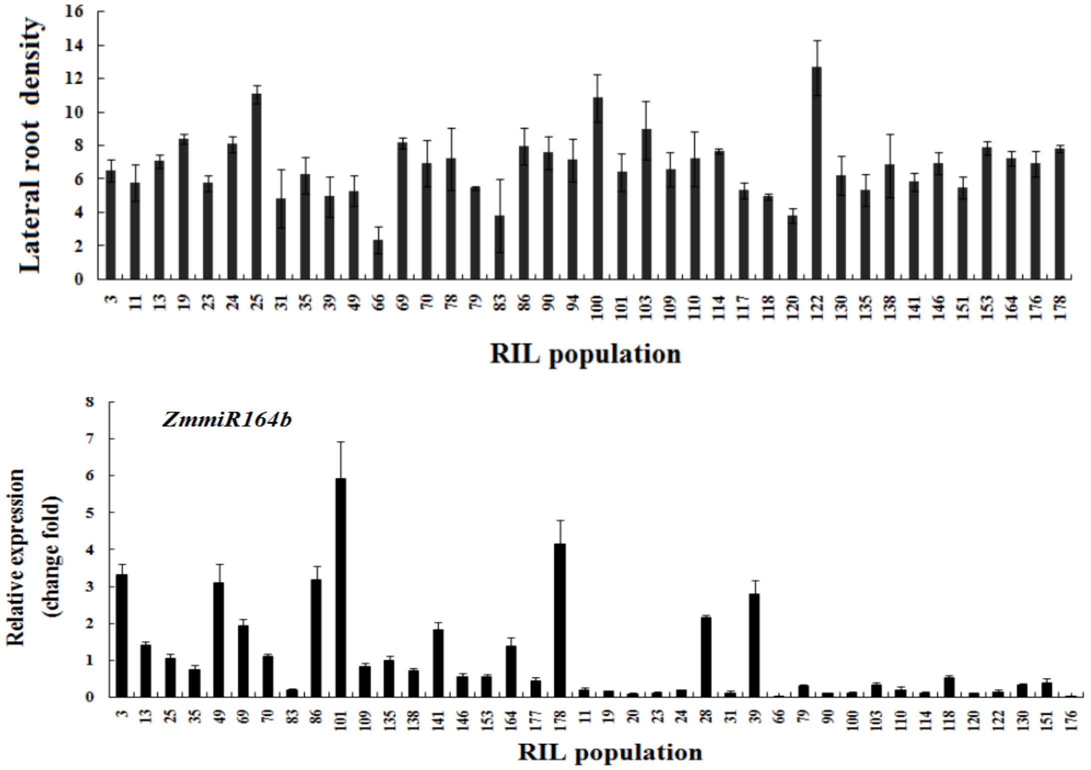

Additional file 7. The correlation between lateral root numbers and expression of mir164b in 40 RILs

This figure shows that a large variation exists in both lateral root numbers and the expression of miR164b among 40 RILs. The correlation coefficient value was calculated, and no significant correlation was found.
