# Supplementary material for: miRNA164-directed cleavage of ZmNAC1 confers lateral root development in maize (Zea mays L.)
Source: BMC Plant Biol. 2012 Nov 21;12:220. doi: 10.1186/1471-2229-12-220 (PMC3554535; doi:10.1186/1471-2229-12-220)
Supplement: Additional file 8 — Comparison of theZmNAC1promoter between 87-1 and Zong3. This figure shows that no large variation exists in the 1 kB region of the ZmNAC1 promoter between two inbred lines, with the exception of one SNP. [file 1471-2229-12-220-S8.pdf]

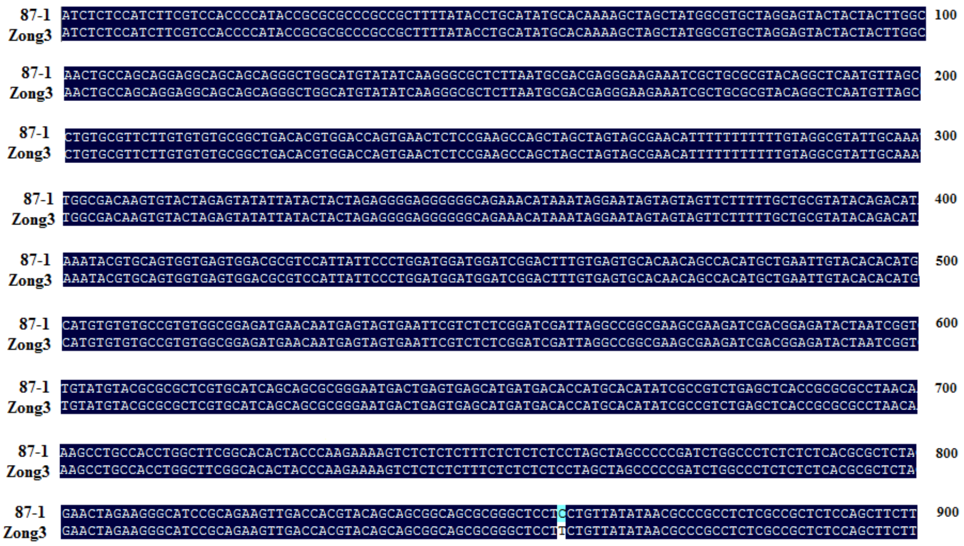

Additional file 8. Comparison of the *ZmNAC1* promoter between 87-1 and Zong3

This figure shows that no large variation exists in the 1 kB region of the *ZmNAC1* promoter between two inbred lines, with the exception of one SNP.
